# A Case of Neural Tube Defect in Early Pregnancy Following Sulfasalazine Exposure Without Folic Acid Supplementation

**DOI:** 10.1155/crrh/8352167

**Published:** 2026-05-29

**Authors:** Francesca Holden, Rodney Hughes

**Affiliations:** ^1^ Rheumatology Department, Ashford and St Peter’s Hospitals NHS Foundation Trust, Surrey, UK

**Keywords:** case report, folic acid, neural tube defect, pregnancy, sulfasalazine

## Abstract

**Background:**

Sulfasalazine is a widely used disease‐modifying antirheumatic drug (DMARD) for inflammatory arthritis and is generally considered safe in pregnancy when co‐prescribed with folic acid. However, its antifolate effects may influence folate metabolism, which is a recognised pathway associated with neural tube defects, particularly when folate supplementation is inadequate.

**Case Presentation:**

A 34‐year‐old woman conceived shortly after starting sulfasalazine without folic acid supplementation. Her pregnancy was complicated by fetal spina bifida and ventriculomegaly detected at 20 weeks’ gestation, leading to termination. She had borderline‐low serum folate before conception (3.7 μg/L). No other teratogenic exposures were identified.

**Conclusion:**

This case highlights the importance of preconception counselling and adherence to high‐dose folic acid (5 mg daily) in women of childbearing potential receiving sulfasalazine. While causality cannot be inferred, it draws attention to a potentially modifiable factor within the multifactorial aetiology of neural tube defects. It also reinforces that medications regarded as safe in pregnancy may still be associated with preventable risk when recommended supplementation is omitted.

**Learning Points**
• Sulfasalazine interferes with folate metabolism but is generally considered safe in pregnancy with appropriate supplementation.
• Women of childbearing potential prescribed sulfasalazine should receive preconception counselling and high‐dose folic acid (5 mg daily).
• Although neural tube defects are multifactorial in origin, sulfasalazine may be associated with a potentially modifiable increased risk in the absence of high‐dose folic acid supplementation.
• Careful documentation and multidisciplinary communication are essential to minimise preventable risks.

## 1. Introduction

Sulfasalazine is a DMARD frequently used in the management of inflammatory arthritis. It is one of the few DMARDs regarded as relatively safe in pregnancy and breastfeeding when appropriately supplemented with folic acid [1, 2]. It interferes with folate metabolism through inhibition of intestinal folate absorption and enzymatic pathways [3].

Neural tube defects (NTDs) are congenital malformations arising from failure of neural tube closure between days 21 and 28 postconception [4]. The global prevalence of NTDs ranges from 1 to 10 per 1000 births, depending on geographical and ethnic factors [4, 5]. Folate status is a well‐established, modifiable risk factor for NTDs, although not the sole determinant, as NTDs arise from a multifactorial interplay of genetic, nutritional, and environmental influences. [4, 5].

Current British Society for Rheumatology guidance recommends high‐dose folic acid (5 mg daily) for women taking sulfasalazine who are planning pregnancy or are pregnant [2]. Despite increasing awareness, inadvertent omission of supplementation continues to occur. We present a case of NTD in a pregnancy occurring shortly after sulfasalazine initiation without folate supplementation, highlighting clinical and preventative considerations such as the importance of preconception counselling and proactive folate replacement.

## 2. Case Presentation

A 34‐year‐old woman was referred to the Rheumatology Department with widespread arthralgia, fatigue and stiffness affecting her elbows, ankles and cervical spine. There was no swelling, rash or systemic features. Her medical history included psoriasis (managed topically) and endometriosis under gynaecological review.

Obstetric history comprised two prior pregnancies: a preterm vaginal delivery at 36 weeks in 2009 complicated by fetal gastroschisis and an emergency caesarean section in 2010 complicated by postpartum haemorrhage requiring transfusion.

Baseline investigations demonstrated normal inflammatory markers (CRP < 1 mg/L, ESR 6 mm/h) and negative rheumatoid factor, anti‐CCP, ANA and HLA‐B27. Ultrasound of the elbows and ankles showed small olecranon bursal effusions without synovitis. MRI of the spine revealed minor degenerative changes at C5/C6 but no evidence of inflammatory spondyloarthropathy. Serum folate was borderline low at 3.7 μg/L (reference range > 4 μg/L).

A provisional diagnosis of psoriatic arthritis was made, and she was initially treated with NSAIDs, which she could not tolerate. In February 2024, she received an intramuscular injection of methylprednisolone acetate (Depo‐Medrone 120 mg) and was commenced on sulfasalazine, titrated from 500 mg daily to 1 g twice daily. Folic acid supplementation was not discussed or prescribed at that stage.

By March 2024, she reported minimal improvement. A concurrent diagnosis of fibromyalgia was made, and sulfasalazine was continued pending review.

In June 2024, she was seen in the antenatal clinic at 13 weeks + 4 days, indicating that conception occurred after initiation of sulfasalazine therapy, although the precise timing relative to folate status during early embryogenesis is uncertain.

At her 20 week anomaly scan, fetal spina bifida and ventriculomegaly were identified. Multidisciplinary counselling was undertaken, and the pregnancy was terminated following fetocide. Placental histopathology was normal. No other potential teratogenic exposures were identified. She did not restart sulfasalazine subsequently.

For any future pregnancy, the patient was advised to take 5 mg of folic acid daily preconception and until 12 weeks’ gestation and will receive early specialist obstetric review and targeted fetal anomaly screening.

## 3. Discussion

This case describes a pregnancy affected by a NTD in the context of recent maternal sulfasalazine exposure without folic acid supplementation and a borderline‐low preconception folate level.

Impaired folate absorption associated with sulfasalazine may have been a contributing factor within a broader multifactorial aetiology. However, NTDs arise from multiple interacting influences, and folate deficiency is recognised as a risk factor rather than a direct cause [4, 5]. Genetic susceptibility and other environmental factors were not assessed in this case and may also have contributed.

Although sulfasalazine is known to impair folate absorption and metabolism, the degree of folate depletion in this patient during early pregnancy is unknown, as folate levels were not re‐measured after treatment initiation or during gestation [3, 6]. Therefore, it cannot be determined whether clinically significant folate deficiency was present at the critical period of neural tube development.

Folate‐dependent one‐carbon metabolism plays a central role in embryogenesis, supporting both nucleotide biosynthesis required for DNA replication and cellular proliferation and methylation reactions involving DNA, RNA, proteins and lipids. These processes collectively contribute to normal cellular growth and differentiation during early development, and disruption of folate‐dependent pathways has been implicated in impaired neural tube closure based on experimental and epidemiological evidence [4, 5].

Epidemiological data do not demonstrate a consistent association between sulfasalazine use and NTDs. While some studies have examined folate‐antagonist medications more broadly, evidence specifically linking sulfasalazine to increased NTD risk is limited, and prior studies have not shown a clear association [7, 8]. Reported associations between sulfasalazine exposure and adverse pregnancy outcomes may be influenced by confounding by indication, including underlying maternal disease activity and concomitant medication use, which may not be fully accounted for in observational studies. Therefore, it remains possible that the NTD in this pregnancy was unrelated to sulfasalazine exposure.

Evidence from animal models has suggested potential differences in the susceptibility of NTD subtypes to folate deficiency, with some studies indicating greater sensitivity of cranial defects such as anencephaly. However, in human populations, most epidemiological and interventional studies have evaluated NTDs as a combined outcome, and robust subtype‐specific associations between folate status and individual defect types (including spina bifida and anencephaly) remain incompletely defined. Current evidence therefore supports folic acid as a broad preventive intervention across the spectrum of NTDs rather than a mechanism that can be clearly stratified by anatomical subtype [4].

Given the background incidence of NTDs, this case may represent a coincidental occurrence. However, the absence of recommended folic acid supplementation represents a potentially modifiable risk factor, particularly in a patient with borderline baseline folate status.

This case therefore does not establish causation but underscores an important clinical principle: even medications considered safe in pregnancy may be associated with risk under certain circumstances, particularly when guideline‐recommended supplementation is omitted.

Preconception counselling should therefore be an integral component of care. When prescribing sulfasalazine to women of childbearing potential, high‐dose folic acid (5 mg daily) should be initiated at least 3 months before conception and continued through the first trimester [2].

It also reinforces the importance of public health strategies such as mandatory folic acid fortification of staple foods to reduce population‐level risk of NTDs [9, 10].

## Consent

Written informed consent was obtained from the patient for publication of this case report and any accompanying details.

## Conflicts of Interest

The authors declare no conflicts of interest.

## Supporting Information

Additional supporting information can be found online in the Supporting Information section.

## Supporting information


**Supporting Information** The CARE checklist for this case report is available as supporting information.

## Data Availability

No underlying datasets were generated or analysed for this case report. All data relevant to the case are contained within the article.
