# Supplementary material for: A Case of Neural Tube Defect in Early Pregnancy Following Sulfasalazine Exposure Without Folic Acid Supplementation
Source: Case Rep Rheumatol. 2026 May 29;2026:8352167. doi: 10.1155/crrh/8352167 (PMC13239456; doi:10.1155/crrh/8352167)
Supplement: Supplementary file 1 — Supporting Information The CARE checklist for this case report is available as supporting information. [file CRRH-2026-8352167-s001.docx]

#
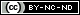

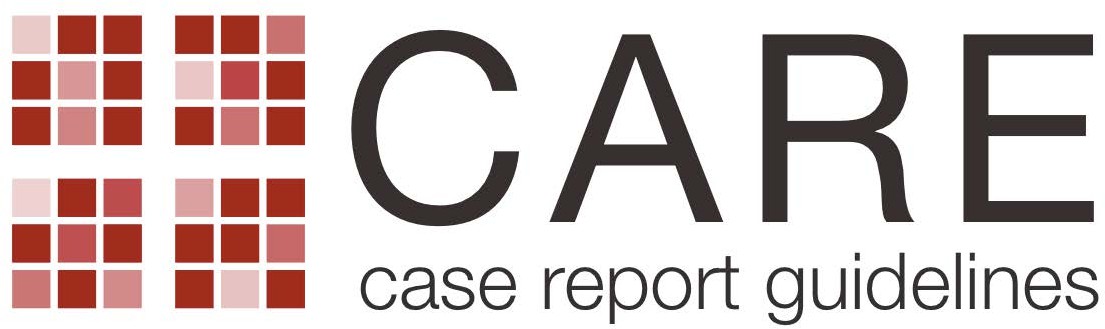
CARE Checklist of information to include when writing a case report

**Topic Item Checklist item description Reported on Line**

**Title 1** The diagnosis or intervention of primary focus followed by the words “case report” . . . . . . . . . . . . . . . . . . Line 1

**Key Words 2** 2 to 5 key words that identify diagnoses or interventions in this case report, including "case report" Line 5

**Abstract**

**(no references)**

**3a** Introduction: What is unique about this case and what does it add to the scientific literature? Lines 7-11

**3b** Main symptoms and/or important clinical findings . . . . . . . . . . . . . . . . . . . . . . . . . . . . . . . . . . . . . . . . . . . . . . . . . . . Lines 12-16

**3c** The main diagnoses, therapeutic interventions, and outcomes Lines 12-16

**3d** Conclusion—What is the main “take-away” lesson(s) from this case? Lines 18-23

**Introduction 4** One or two paragraphs summarizing why this case is unique (**may include** reference**s**) Lines 24-40

**Patient Information 5a** De-identified patient specific information Lines 43-71

**5b** Primary concerns and symptoms of the patient Lines 43-45

**5c** Medical, family, and psycho-social history including relevant genetic information Lines 45-49

**5d** Relevant past interventions with outcomes Lines 45-49

**Clinical Findings**

**Timeline**

**Diagnostic Assessment**

**Therapeutic Intervention**

**Follow-up and Outcomes**

1. Describe significant physical examination (PE) and important clinical findings Lines 44-45
2. Historical and current information from this episode of care organized as a timeline Chronology described narratively within Case Presentation (lines 42-70)

**8a** Diagnostic testing (such as PE, laboratory testing, imaging, surveys). Lines 50-54

**8b** Diagnostic challenges (such as access to testing, financial, or cultural) None identified

**8c** Diagnosis (including other diagnoses considered) Line 65

**8d** Prognosis (such as staging in oncology) where applicable Not applicable; pregnancy outcome determined following prenatal diagnosis

**9a** Types of therapeutic intervention (such as pharmacologic, surgical, preventive, self-care) …………………………….Lines 57-59 & 66-67

**9b** Administration of therapeutic intervention (such as dosage, strength, duration) Lines 57-59

**9c** Changes in therapeutic intervention (with rationale) Lines 68-71

**10a** Clinician and patient-assessed outcomes (if available) Fetal neural tube defect identified at 20-week scan leading to termination of pregnancy following multidisciplinary counselling (Lines 65-71)

**10b** Important follow-up diagnostic and other test results Placental histopathology reported as normal (Lines 67-68)

**10c** Intervention adherence and tolerability (How was this assessed?) Not formally assessed. Sulfasalazine was tolerated but prescribed without folic acid supplementation

**10d** Adverse and unanticipated events Fetal neural tube defect (spina bifida with ventriculomegaly) identified during pregnancy.

**Discussion 11a** A scientific discussion of the strengths AND limitations associated with this case report Lines 72-118

**11b** Discussion of the relevant medical literature **with references** Lines 72-118 with references lines 139-159

**11c** The scientific rationale for any conclusions (including assessment of possible causes) Lines 72-118

**11d** The primary “take-away” lessons of this case report (without references) in a one paragraph conclusion Lines 111-118 and learning point summary lines 120-129

**Patient Perspective 12** The patient should share their perspective in one to two paragraphs on the treatment(s) they received . . . .A patient perspective was not included due to the sensitive nature of the case and to preserve patient privacy.

**Informed Consent 13** Did the patient give informed consent? Please provide if requested . . . . . . . . . . . . . . . . . . . . . . . . . . . . . . . . . . . . . **Yes**
